# Four new species of the genus *Lathrolestes* Förster, 1869 from South Korea (Hymenoptera, Ichneumonidae, Ctenopelmatinae)

**DOI:** 10.3897/zookeys.657.11630

**Published:** 2017-02-17

**Authors:** Alexey Reshchikov, Jin-Kyung Choi, Jong-Wook Lee

**Affiliations:** 1 College of Ecology and Evolution, Sun Yat-sen University, 135 Xingangxi St.Guangzhou, Guangdong 510275, China; 2 Department of Life Sciences, Yeungnam University, Gyeongsan, South Korea

**Keywords:** Eastern Palaearctic, Korea, key, Perilissini, parasitoid wasps, taxonomy

## Abstract

Four new species of the genus *Lathrolestes* Förster, 1869 are discovered from South Korea: *Lathrolestes redimiculus* Reshchikov & Lee, **sp. n.**, *Lathrolestes sexmaculatus* Reshchikov & Lee, **sp. n.**, *Lathrolestes taebaeksanensis* Reshchikov & Lee, **sp. n.**, and *Lathrolestes ungnyeo* Reshchikov & Lee, **sp. n.** This is the first record of the genus from South Korea.

## Introduction

Ctenopelmatinae Förster, 1869 comprise more than 1,390 described species within 106 genera, including 373 Eastern Palaearctic species (Yu et al. 2012). The South Korean Ctenopelmatinae were reviewed by Uchida (1955), Kim (1955), Townes et al. (1965), and Townes (1970) and 14 species were reported from South Korea recently (Yu et al. 2012, Choi et al. 2016, Kasparyan et al. 2016). The current total is 38 species of Ctenopelmatinae known from South Korea. The genus *Lathrolestes* Förster, 1869 belongs to the tribe Perilissini subfamily Ctenopelmatinae (Ichneumonidae) and comprises 105 previously described distinct species (Yu et al. 2012, Reshchikov 2015a, 2015b, Lima and Kumagai 2016) including 21 species distributed in the Eastern Palaearctic (Reshchikov 2012a, 2012b). In the present paper, four new species from South Korea are considered: *Lathrolestes redimiculus* sp. n., *Lathrolestes sexmaculatus* sp. n., *Lathrolestes taebaeksanensis* sp. n., and *Lathrolestes ungnyeo* sp. n. This is the first record of the genus from South Korea. It is significant that no previously known Eastern Palaearctic species have been found within the rather representative material examined. In this paper, descriptions of four new species and a key to the species of South Korean *Lathrolestes* are provided.

## Materials and methods

Materials used in this study were collected by sweeping (*Lathrolestes redimiculus* sp. n. and *Lathrolestes ungnyeo* sp. n.) and Malaise trapping (M.T.: *Lathrolestes sexmaculatus* sp. n. and *Lathrolestes taebaeksanensis* sp. n.). The morphological terminology follows that of Gauld (1991). Photographs were taken at the Department of Life Sciences, Yeungnam University, Gyeongsan-si, Gyeongsangbuk-do, Republic of Korea (YNU) with an AxioCam MRc5 camera attached to a stereo microscope (Zeiss SteREO Discovery. V20; Carl Zeiss, Göttingen, Germany), processed using AxioVision SE64 software (Carl Zeiss), and optimized with a Delta imaging system (i-solution, IMT I Solution Inc. Vancouver, Canada). The type specimens of the four new species are deposited in YNU. Abbreviations are used as follows: **CB**: Chungcheongbuk-do; **GB**: Gyeongsangbuk-do; **GG**: Gyeonggi-do; **GW**: Gangwon-do.

## Taxonomy

### Family Ichneumonidae Latreille, 1802

#### Subfamily Ctenopelmatinae Förster, 1869

##### Tribe Perilissini Thomson, 1883

###### Genus Lathrolestes

Taxon classificationAnimaliaHymenopteraIchneumonidae

Förster, 1869

Lathrolestes Förster, 1869: 135–221. Type species: Tryphon clypeatus.
Camporychus Förster, 1869: 135–221. Type species: Lathrolestus marginatus.
Ecclinops Förster, 1869: 135–221. Type species: Tryphon orbitalis.
Homalomma Förster, 1869: 135–221. Type species: Homalomma caliroae.
Lathrolestus Thomson, 1883: 873–936. Type species: Tryphon clypeatus.
Luphyroscopus Thomson, 1883: 873–936. Type species: Tryphon gorskii.
Tryphonopsis Brauns, 1898: 58–72. Type species: Tryphonopsis ensator.
Ritzemabosia Smits van Burgst, 1912: 263–270. Type species: Ritzemabosia meridionalis.

####### Diagnosis.

Small to medium sized species, 4.0–7.5 mm. Occipital carina not intercepting hypostomal carina. Clypeus profile flat, its apical margin thick. Mandible with lower tooth distinctly longer than upper. Areolet petiolate, oblique; vein 2m-cu of fore wing with single bulla; vein cu-a of hind wing intercepted below or at its middle. Tarsal claws pectinate, with 1 or 3 teeth or with basal lobe. Glymmae deep. Apex of subgenital plate of male not incurved on posterior margin. Tip of aedeagus somewhat decurved and swollen, its apex rounded. Ovipositor sheath 0.3 to 15 × as long as metasomal height.

####### Key to species of *Lathrolestes* occurring in South Korea

**Table:** 

1	Apical margin of clypeus truncated. Malar space short, less than 0.5 × basal mandible width. Ovipositor straight, without notch. Hind wing with cu-a intercepted by Cu1 in the middle	***Lathrolestes ungnyeo* sp. n.**
–	Apical margin of clypeus thick and rounded. Malar space more than 0.5 × basal mandible width. Ovipositor straight, stout, with rather shallow notch or shallow impression (except for *Lathrolestes redimiculus*, female unknown). Hind wing with cu-a intercepted by Cu1 below middle	**2**
2	First tergite more than 1.7 × as long as broad apically, without longitudinal dorsal carinae. Head not narrowed eyes posteriorly. Mesoscutum with notaulus not defined	***Lathrolestes taebaeksanensis* sp. n.**
–	First tergite less than 1.5 × as long as broad apically, with longitudinal dorsal carinae completely or basally. Head narrowed eyes posteriorly. Mesoscutum with notaulus shallow	**3**
3	Clypeus distinctly separated from face. Areolet petiolate. Propodeal carinae complete	***Lathrolestes sexmaculatus* sp. n.**
–	Clypeus not separated from face. Areolet not petiolate. Propodeum with only apical carina complete	***Lathrolestes redimiculus* sp. n.**

###### Lathrolestes redimiculus

Taxon classificationAnimaliaHymenopteraIchneumonidae

Reshchikov & Lee sp. n.

http://zoobank.org/1EB54BA7-E408-465C-A8D6-90B4AAC2F687

Figure 1

####### Diagnosis.

**Figure 1.**
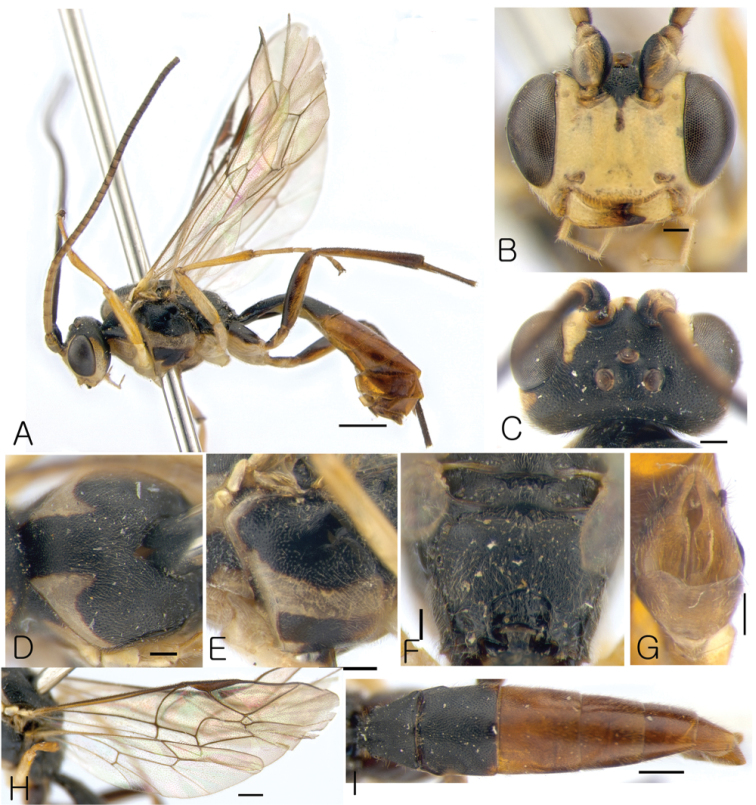
*Lathrolestes redimiculus*; **A** Habitus in lateral view **B** Head in anterior view **C** Head in dorsal view **D** Mesoscutum in dorsal view **E** Mesopleuron **F** Propodeum **G** Genitalia **H** Wings **I** Metasoma in dorsal view. Scale bars: 1 mm (**A**); 0.5 mm (**E, I**); 0.2 mm (**B–D, F–H**).

This species is similar to *Lathrolestes verticalis* (Brischke, 1871) but distinguishable by combination of the following characters: clypeus not separate from face by distinct groove (Fig. 1B); mesopleuron and metasoma distinctly punctate (Fig. 1E, I); areolet of fore wing closed (Fig. 1H); area petiolaris of propodeum complete; tergites 1 and 2 entirely black; further tergites reddish (Fig. 1I).

####### Description.

Male. Body length 7.7 mm.

*Head*. Matt, punctate with shallow and sparse punctures on shagreen surface (Fig. 1C). Face 1.15 × as broad as eye height, relatively flat, bulging (Fig. 1B). Clypeus not separated from face, at apex projecting anteriorly (Fig. 1B); apical margin of clypeus thick. Clypeal fovea large. Malar space 0.6 × as long as basal mandible width. Lower mandible tooth longer than upper. Occipital carina complete.

*Mesosoma*. Matt. Pronotum punctate with sparse punctures. Epomia absent. Mesoscutum punctate with shallow indistinct punctures, matt, with notaulus shallow (Fig. 1D). Mesopleuron distinctly and sparsely punctate with fine punctures, polished (Fig. 1E). Claws not pectinate. Fore wing with R intercepting pterostigma before its middle (Fig. 1H); areolet not petiolate; vein 2m-cu of fore wing with single bulla. Vein cu-a of hind wing slightly postfurcal, with cu-a intercepted by Cu1 below middle. Propodeum with only apical carina complete (Fig. 1F).

*Metasoma*. Matt, distinctly and densely punctate with shallow punctures (Fig. 1I). Tergite 1 1.5 × as long as broad apically, convex, with longitudinal dorsal carinae and medial impression (Fig. 1I). Tergite 2 rectangle (Fig. 1I). Following tergites also elongate (Fig. 1I). Parameres broad at base (Fig. 1G).

*Color*. Body mostly black. Scape, antennal flagellum ventrally, face entirely, temple eye posteriorly, mandible except teeth, propleurum, lateral parts of mesonotum, tegula, band in the middle of mesopleurum, fore and middle legs, hind coxa ventrally yellowish. Hind femur ventrally, hind tibia basally, metasomal starting at tergite 3 reddish.

***Female***. Unknown.

**Etymology**. The name *redimiculus* refers to yellow band of the middle of mesopleurum.

**Material examined**. Holotype: male; type depository: YNU, GW, Hangrobong (Sweeping), 13.vi.1992, leg. S.M. Ryu. Paratypes: 3 males, GW, Hangrobong, (Sweeping), 13.vi.1992, leg. S.M. Ryu (YNU); 1 male, ditto, (Sweeping), leg. J.W. Lee (YNU).

###### Lathrolestes sexmaculatus

Taxon classificationAnimaliaHymenopteraIchneumonidae

Reshchikov & Lee sp.n.

http://zoobank.org/0E01E180-5FE8-4F28-865E-0A98A0C9C105

Figure 2

####### Diagnosis.

This species is similar to *Lathrolestes grahami* Reshchikov, 2012 and *Lathrolestes tolstoyi* Reshchikov, 2012 but distinguishable by combination of the following characters: claws not pectinate, malar space 0.9 × as long as basal mandible width; face in female black with small yellow macula between antennal socket and eye margin, ventral part of eye, lateral edge of clypeus, face in male mostly yellow with black bands ventral part of antennal sockets, malar space and middle apical part of clypeus.

####### Description.

Female. Body length 7.3 mm.

**Figure 2.**
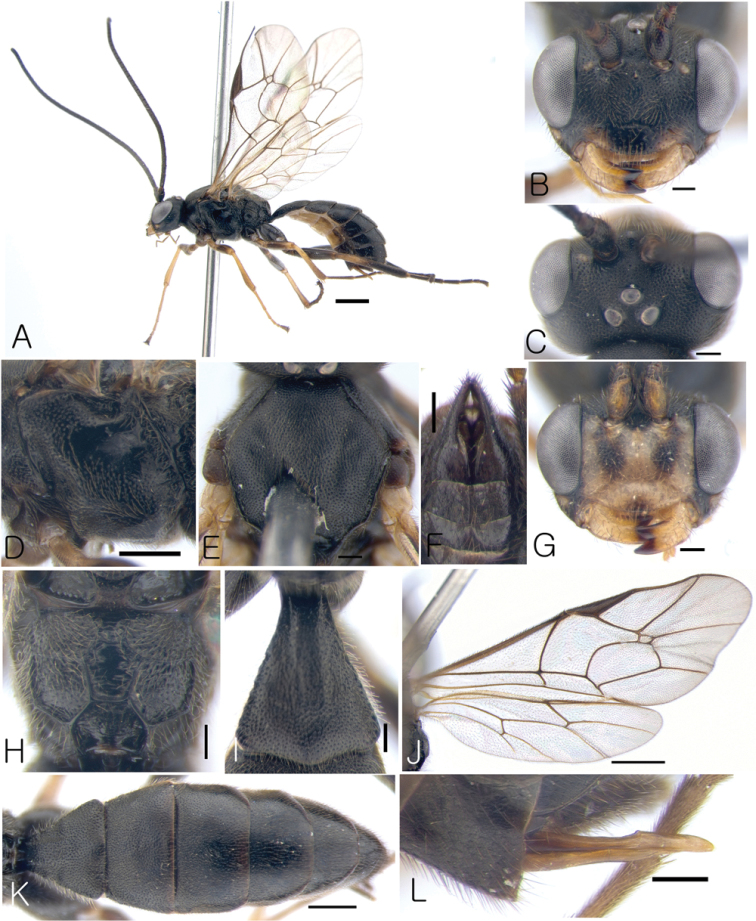
*Lathrolestes sexmaculatus* (Female except **F, G**); **A** Habitus in lateral view **B** Head in anterior view **C** Head in dorsal view **D** Mesopleuron **E** Mesoscutum in dorsal view **F** Genitalia of male **G** Head in anterior view **H** Propodeum **I** First tergite in dorsal view **J** Wings **K** Metasoma in dorsal view **L** Ovipositor. Scale bars: 1 mm (**A, J**); 0.5 mm (**D, K**); 0.2 mm (**B, C, E–I, L**).

*Head*. Matt, distinctly punctate with fine and dense punctures on shagreen surface (Fig. 2C). Face 1.3 × as broad as eye height, relatively flat, bulging (Fig. 2B). Clypeus distinctly separated from face by groove, at apex projecting anteriorly (Fig. 2B); apical margin of clypeus thick. Clypeal fovea relatively small. Malar space 0.9 × as long as basal mandible width. Lower mandible tooth almost equal to upper. Occipital carina complete.

*Mesosoma*. Matt. Pronotum distinctly punctate. Epomia absent. Mesoscutum distinctly punctate, matt, with notaulus shallow. (Fig. 2E). Mesopleuron finely and densely punctate, polished (Fig. 2D). Claws not pectinate. Fore wing with R intercepting pterostigma before its middle (Fig. 2J); areolet petiolate; vein 2m-cu of fore wing with single bulla. Vein cu-a of hind wing interstitial, with cu-a intercepted by Cu1 below middle. Propodeal carinae complete (Fig. 2H).

*Metasoma*. Matt, distinctly and densely punctate (Fig. 2K). Tergite 1 1.2 × as long as broad apically, convex, with longitudinal dorsal carinae distinct basally and medial impression (Fig. 2I). Tergite 2 transverse, trapezoidal (Fig. 2K). Following tergites also transverse. Ovipositor straight, stout, with rather shallow notch in the middle (Fig. 2L).

*Color*. Body mostly black (Figs 2A–K). Mandible except teeth, lateral edges of clypeus, maculae ventral eye and between eye and antennal sockets, fore tibia and tarsus except apical tarsal segment, basal half of middle tibia, and basal part of hind tibia yellowish (Fig. 2A)

***Male*.** Body length 7 mm. Character states are mostly the same as in female. Parameres elongate (Fig. 2F).

*Color*. Same as in female but scape and face mostly yellow (with band ventral part of each scape, tip of clypeus and malar space black) (Fig. 2G).

####### Etymology.

Name *sexmaculatus* refers to six yellow maculae on head in female.

####### Material examined.

Holotype: female; type depository: YNU, CB Danyang-gun, Danyang-eup, Cheongdong-ri, 35°57'N 128°28'E (M.T.), 8.vi–6.vii.2005, leg. J.W. Lee. Paratypes: 1 female 1 male, GG Yangpyeong-gun, Yongmun-myeon, Yeonsu-ri, Mt. Yongmunsan, 37°31'49.5"N 127°34'18.8"E (M.T.), Alt. 324 m, 11–25.vi.2009, leg. J.O. Lim (YNU); 1 female, CB Danyang-gun, Danyang-eup, Cheongdong-ri, 35°57'N 128°28'E (M.T.), 8.vi–6.vii.2005, leg. J.W. Lee (YNU).

###### Lathrolestes taebaeksanensis

Taxon classificationAnimaliaHymenopteraIchneumonidae

Reshchikov & Lee sp. n.

http://zoobank.org/ED651E22-89DE-48B8-832A-C65CDBA96FFC

Figure 3

####### Diagnosis.

**Figure 3.**
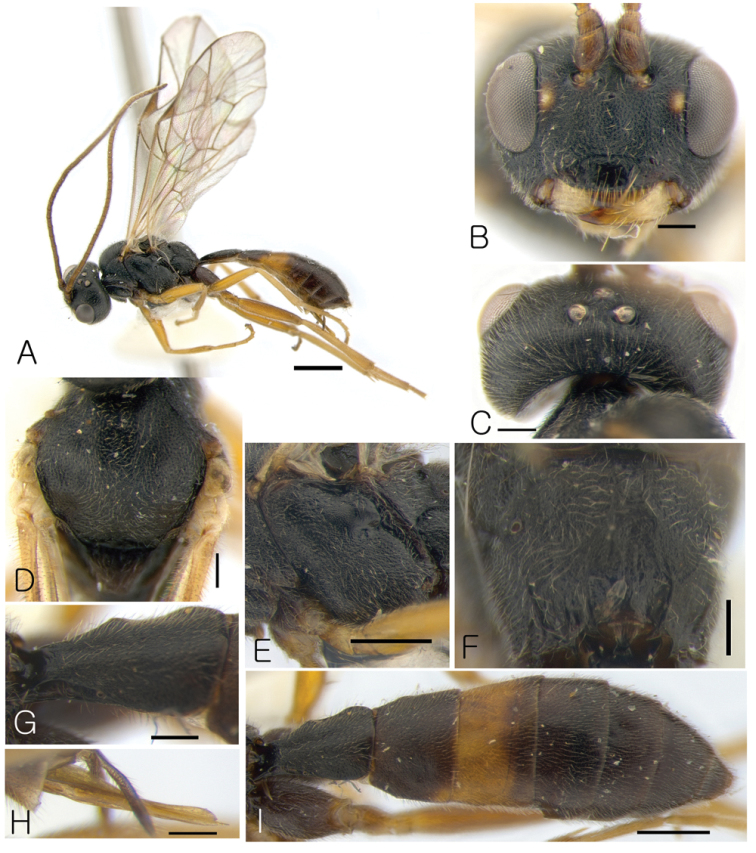
*Lathrolestes taebaeksanensis*: **A** Habitus in lateral view **B** Head in anterior view **C** Head in dorsal view **D** Mesoscutum in dorsal view **E** Mesopleuron **F** Propodeum **G** First tergite in dorsal view **H** Ovipositor **I** Metasoma in dorsal view. Scale bars: 1 mm (**A**); 0.5 mm (**E, I**); 0.2 mm (**B–D, F–H**).

This species generally is similar to *Lathrolestes soperi* Reshchikov, 2010, and its ovipositor structure is similar to those of *Lathrolestes breviremus* Barron, 1994, *Lathrolestes erugatus* Barron, 1994, and *Lathrolestes tolstoyi* Reshchikov, 2012 but distinguishable by combination of the following character states: claw simple, yellow maculae between antennal sockets and eye margin little ventral level of antennal sockets, malar space as long as basal mandible width, face 1.5 × as broad as eye, height (Fig. 3B), notaulus not defined (Fig. 3D), propodeum with carinae obliterated (Fig. 3F), tergite 1 without dorsal longitudinal carinae (Fig. 3G), ovipositor straight, stout at base, upper valve rounded and at tip with shallow impression in the middle (Fig. 3H).

####### Description.

Female. Body length 5.3 mm.

*Head*. Matt, not punctate, shagreen (Fig. 3C). Face 1.5 × as broad as eye height, projecting in the middle, bulging (Fig. 3B). Clypeus distinctly separated from face by groove, at apex projecting anteriorly (Fig. 3B); apical margin of clypeus thick. Clypeal fovea small. Malar space as long as basal mandible width. Lower mandible tooth longer than upper. Occipital carina complete.

*Mesosoma*. Matt. Pronotum not punctate. Epomia absent. Mesoscutum finely punctate, matt, with notaulus not defined (Fig. 3D). Mesopleuron finely and sparsely punctate, shagreen (Fig. 3E). Claws not pectinate. Fore wing with R intercepting pterostigma at its middle; areolet not petiolate; vein 2m-cu of fore wing with single bulla. Vein cu-a of hind wing interstitial, with cu-a intercepted by Cu1 below middle. Propodeal carinae oblitirated (Fig. 3F).

*Metasoma*. Matt, not punctate (Fig. 3I). First metasomal tergite 1.94 × as long as broad apically, convex, without longitudinal dorsal carinae (Fig. 3G). Second metasomal tergite transverse, trapezoidal (Fig. 3I). Following tergites also transverse. Ovipositor straight, stout at base and roundish at tip with shallow impression in the middle (Fig. 3H).

*Color*. Body mostly black (Fig. 3A–I). Maculae between antennal sockets and eye margin little ventral level of antennal sockets, mandible except teeth, tegula, legs except coxae and hind femur yellowish (Fig. 3A, B, D). Antenna, hind femur and tergite 3 mostly reddish (Fig. 3I).

Male. Unknown.

####### Etymology.

The name *taebaeksanensis* refers to Mt. Taebaeksan where the species was collected.

####### Material examined.

Holotype: female; type depository: YNU, GW Mt. Taebaeksan National Park (M.T.), 14.v–20.vi.1999, D.S. Gu.

###### Lathrolestes ungnyeo

Taxon classificationAnimaliaHymenopteraIchneumonidae

Reshchikov & Lee sp. n.

http://zoobank.org/CD54711E-BC3D-4CF0-B49D-0D117977A784

Figure 4

####### Diagnosis.

**Figure 4.**
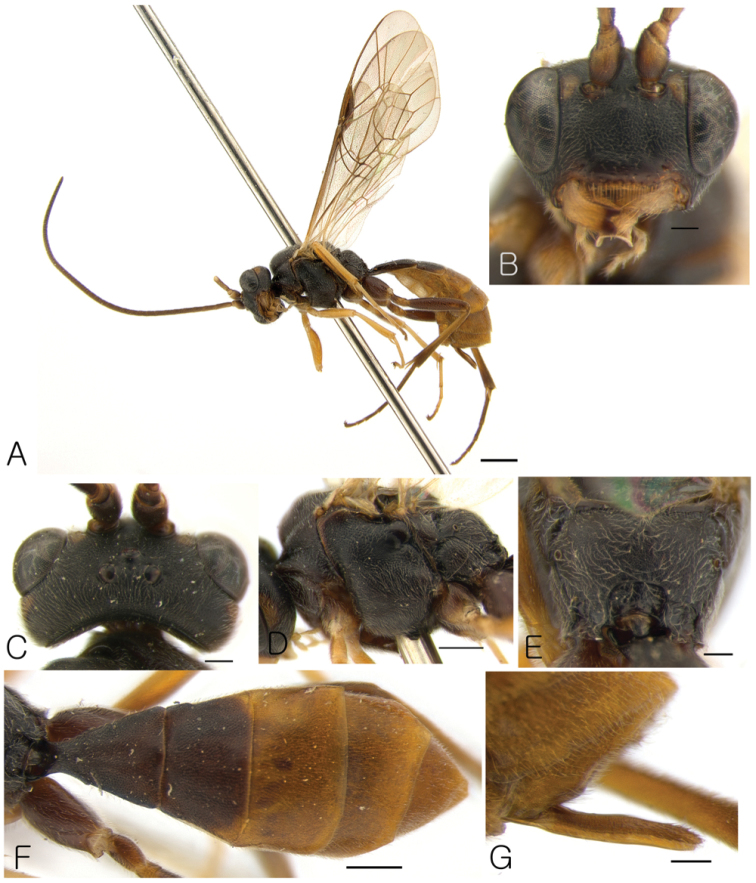
*Lathrolestes ungnyeo*: **A** Habitus in lateral view **B** Head in anterior view **C** Head in dorsal view **D** Mesopleuron **E** Propodeum; **F** Metasoma in dorsal view **G** Ovipositor sheath. Scale bars: 1 mm (**A**); 0.5 mm (**D, F**); 0.2 mm (**B, C, E, G**).

This species similar to *Lathrolestes cruentocaudus* Reshchikov, 2012, *Lathrolestes palatynus* Reshchikov, 2012, *Lathrolestes redimiculus* sp. n., and *Lathrolestes verticalis* (Brischke, 1871) but distinguishable by combination of the following character states: clypeus not separate from face, not projecting anteriorly, apically truncated (Fig. 4B), hind wing with cu-a intercepted by Cu1 in the middle (Fig. 4A), tergite 1 without longitudinal dorsal carinae, ovipositor straight, without notch (Fig. 4F).

####### Description.

Female. Body length 7.5 mm.

*Head*. Matt, not punctate, shagreen (Fig. 4C). Face 1.2 × as broad as eye height, flat, bulging (Fig. 4B). Clypeus not separated from face, not projecting anteriorly (Fig. 4B), apically truncated (Fig. 4B). Clypeal fovea small. Malar space 0.46 × as long as basal mandible width. Lower mandible tooth almost equal to upper. Occipital carina complete.

*Mesosoma*. Matt. Pronotum not punctate. Epomia absent. Mesoscutum finely punctate, matt, with notaulus not defined. Mesopleuron finely and sparsely punctate, shagreen (Fig. 4D). Claws not pectinate. Fore wing with R intercepting pterostigma before the middle (Fig. 4A); areolet not petiolate; vein 2m-cu of fore wing with single bulla. Vein cu-a of hind wing interstitial, with cu-a intercepted by Cu1 in the middle. Propodeum with only apical carina complete (Fig. 4E).

*Metasoma*. Matt, not punctate (Fig. 4F). Tergite 1 1.35 × as long as broad apically, convex, without longitudinal dorsal carinae (Fig. 4F). Tergite 2 transverse, trapezoidal (Fig. 4F). Following tergites also transverse. Ovipositor straight (Fig. 4G).

*Color*. Body mostly black (Fig. 4A–F). Maculae between antennal sockets and eye margin, ventral part of antenna, apical margin of clypeus, mandible except teeth, tegula, fore leg entirely, middle tibia and tarsus yellowish (Figs 4B, 4D). Middle femur and metasoma starting at tergite 3 reddish (Fig. 4F). Hind leg brownish (Fig. 4A)

Male. Unknown.

**Figure 5.**
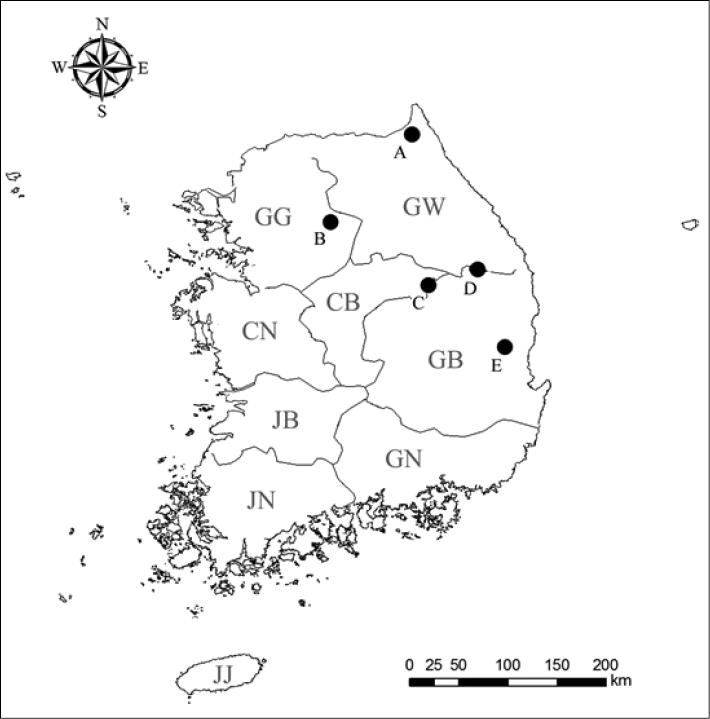
Distribution map of South Korean *Lathrolestes* spp.: **A** *Lathrolestes redimiculus* sp. n. **B–C** *Lathrolestes sexmaculatus* sp. n. **D** *Lathrolestes taebaeksanensis* sp. n. **E** *Lathrolestes ungnyeo* sp. n.

####### Etymology.

The name *ungnyeo* refers to the Ungnyeo, or “bear woman” which is the progenitress of Koreans in Korean mythology.

####### Material examined.

Holotype: female; type depository: YNU, GB Cheongsong-gun, Juwangsan National Park (sweeping), 17.v.1987, S.M. Ryu.

## Supplementary Material

XML Treatment for Lathrolestes

XML Treatment for Lathrolestes redimiculus

XML Treatment for Lathrolestes sexmaculatus

XML Treatment for Lathrolestes taebaeksanensis

XML Treatment for Lathrolestes ungnyeo
